# [Corrigendum] Integrative transcriptomic analysis unveils FXR as a key regulator of intestinal stemness and inflammation in ulcerative colitis

**DOI:** 10.3892/ijmm.2026.5985

**Published:** 2026-09-14

**Authors:** Hao Lin, Xinran Cheng, Yangyang Ren, Yongmei Zhang, Weixu Chen, Qingyan Lu, Yiqing Tian

Int J Mol Med 58: 200, 2026; DOI: 10.3892/ijmm.2026.5871

Following the publication of the above article, an interested reader raised several comments on the study that required clarification on the part of the authors in order to confirm the reporting of certain of the results, and to make the paper fully comprehensible. First, in the Materials and methods section, the single-cell RNA sequencing (RNA-seq) source did not appear to match the presented groups for the scRNA-seq data that were obtained from the GSE116222 dataset, specifically using 'three human UC samples', where three distinct groups, namely the 'Normal', 'Non-inflamed' and 'Inflamed' groups, were shown in various of the figures (for example, Fig. 1D and Fig. 6K).

Secondly, the description of the Weighted Gene Co-expression Network Analysis (WGCNA) in the Materials and methods section did not tally with the findings reported in the Results section. It was stated in the '*Construction of co-expression network*' subsection of the Methods that WGCNA was performed 'based on the GSE116222 dataset, specifically exploring the association between FXR and CD133 in intestinal cells'; however, all the WGCNA results shown (in Figs. 4D and 5A) were based on the bulk transcriptome dataset GSE87466, not on single-cell data; no WGCNA output from GSE116222 was presented in the paper.

Thirdly, some confusion between human and mouse data appeared in the reporting of the experiments in Fig. 1E. The figure part showed macroscopic photographs of whole large intestines and spleens labeled 'Control' and 'UC groups', and these images must have been derived from the DSS-treated mouse model. The figure legend did not specify that these are mouse data, and the labeling of the 'UC group' was misleading.

Finally, a typographical error in the reporting of the human control group sex distribution was contained in the '*Human sample collection and processing*' subsection: 'the control group consisted of 8 men and 2 men' was presumably intended to have read as '8 men and 2 women'.

The authors have considered these points, and acknowledge that clarifications of all of them are necessary in the published paper; therefore, the following changes and edits to the text are required (all highlighted in **bold**). First, in the 'Materials and methods' section, in the '*Single-cell RNA sequencing (scRNA-seq)*' subsection, the text which read as 'scRNA-seq data from three human UC samples were downloaded from the Gene Expression Omnibus (GEO) database…' has now been changed to 'scRNA-seq data from **9 samples (3 healthy, 3 non-inflamed, and 3 UC)** were downloaded from the Gene Expression Omnibus (GEO) database…'

In response to the second of the reader's queries, in the '*Construction of co-expression network*' subsection of the Methods, the text which read: 'In the present study, the 'WGCNA' package in R was employed to construct a co-expression network based on the GSE116222 dataset, specifically exploring the association between FXR and CD133 in intestinal cells.', should be changed to: 'In the present study, the 'WGCNA' package in R was employed to construct a co-expression network based on the **GSE87466 dataset to identify key gene modules associated with UC.'**

In response to the third query, and to properly clarify the descriptions of the experiments in the figure legend for Fig. 1, the text for Fig. 1E should be changed from what was written to the following: '(E) Representative macroscopic images of the large intestines and spleens from the Control and **DSS** groups.' (i.e., 'UC groups' has been corrected to 'DSS groups').

Finally, the authors acknowledged that a typographical error was present in the reporting of the human control group sex distribution in the '*Human sample collection and processing*' subsection, and the text here should have read as 'the control group consisted of 8 men and **2 women** (mean age, 46.2±7.5 years).'

The authors thank the reader for drawing these matters to their attention. They are grateful to the Editor of *International Journal of Molecular Medicine* for granting them the opportunity to publish this corrigendum. Note that the proposed changes to the text only correct errors in what was written, or clarify the meaning of the text; the results and conclusions of the paper are not affected by these changes. Finally, the authors apologize to the readership for any inconvenience caused.

